# Mutational Profiling Can Establish Clonal or Independent Origin in Synchronous Bilateral Breast and Other Tumors

**DOI:** 10.1371/journal.pone.0142487

**Published:** 2015-11-10

**Authors:** Lei Bao, Karen Messer, Richard Schwab, Olivier Harismendy, Minya Pu, Brian Crain, Shawn Yost, Kelly A. Frazer, Brinda Rana, Farnaz Hasteh, Anne Wallace, Barbara A. Parker

**Affiliations:** 1 Moores Cancer Center, University of California San Diego, La Jolla, CA, United States of America; 2 Division of Biostatistics, Department of Family Medicine and Public Health, University of California San Diego, La Jolla, CA, United States of America; 3 Department of Medicine, University of California San Diego, La Jolla, CA, United States of America; 4 Department of Pediatrics, University of California San Diego, La Jolla, CA, United States of America; 5 Department of Psychiatry, University of California San Diego, La Jolla, CA, United States of America; 6 Department of Pathology, University of California San Diego, La Jolla, CA, United States of America; 7 Department of Surgery, University of California San Diego, La Jolla, CA, United States of America; University of North Carolina School of Medicine, UNITED STATES

## Abstract

**Background:**

Synchronous tumors can be independent primary tumors or a primary-metastatic (clonal) pair, which may have clinical implications. Mutational profiling of tumor DNA is increasingly common in the clinic. We investigated whether mutational profiling can distinguish independent from clonal tumors in breast and other cancers, using a carefully defined test based on the Clonal Likelihood Score (CLS = 100 x # shared high confidence (HC) mutations/ # total HC mutations).

**Methods:**

Statistical properties of a formal test using the CLS were investigated. A high CLS is evidence in favor of clonality; the test is implemented as a one-sided binomial test of proportions. Test parameters were empirically determined using 16,422 independent breast tumor pairs and 15 primary-metastatic tumor pairs from 10 cancer types using The Cancer Genome Atlas.

**Results:**

We validated performance of the test with its established parameters, using five published data sets comprising 15,758 known independent tumor pairs (maximum CLS = 4.1%, minimum p-value = 0.48) and 283 known tumor clonal pairs (minimum CLS 13%, maximum p-value <0.01), across renal cell, testicular, and colorectal cancer. The CLS test correctly classified all validation samples but one, which it appears may have been incorrectly classified in the published data. As proof-of-concept we then applied the CLS test to two new cases of invasive synchronous bilateral breast cancer at our institution, each with one hormone receptor positive (ER+/PR+/HER2-) lobular and one triple negative ductal carcinoma. High confidence mutations were identified by exome sequencing and results were validated using deep targeted sequencing. The first tumor pair had CLS of 81% (p-value < 10–15), supporting clonality. In the second pair, no common mutations of 184 variants were validated (p-value >0.99), supporting independence. A plausible molecular mechanism for the shift from hormone receptor positive to triple negative was identified in the clonal pair.

**Conclusion:**

We have developed the statistical properties of a carefully defined Clonal Likelihood Score test from mutational profiling of tumor DNA. Under identified conditions, the test appears to reliably distinguish between synchronous tumors of clonal and of independent origin in several cancer types. This approach may have scientific and clinical utility.

## Introduction

Synchronous bilateral breast cancer (SBBC), in which separate tumors are diagnosed simultaneously in each breast, occurs in 1–3% of all breast cancer patients [1], and incidence has increased in the era of MRI screening [2]. The two tumors may be clonal, with one tumor a metastasis of the other, or they may be independent tumors arising spontaneously within the same genetic background. Current standard of care for SBBC is to assume independent origin with curative intent treatment for both tumors, and increased germline risk but not worse prognosis [3, 4]. Therapy decisions are guided by the higher-risk tumor [5]. However, improved understanding of clonal etiology in SBBC may have implications for patient prognosis and familial risk assessment, as well as, for the biology of breast cancer evolution and metastasis.

Prior studies aiming to distinguish clonal from independently arising SBBC tumors have been limited in their ability to detect clonal status. They have used presence of concordant histological features and systemic metastases [6] or been based on concordance among a set of fewer than 20 molecular markers [3, 4, 7–9], with limited statistical power to detect overlaps. Thus, the incidence of clonal SBBC is likely underestimated [10, 11]. In similar studies of ipsilateral breast, lung and other tumor types, newer high resolution array-based approaches have found greater occurrence of clonal tumors than previously appreciated [4, 12–17], and formal statistical tests based on chromosomal copy number aberrations have been developed [10, 11, 13, 16, 18]. On the other hand, mutational profiling of tumor DNA is increasingly common in the clinic. While several recent studies of matched primary tumor and metastasis have investigated use of mutational profiling of tumor DNA to determine clonal status [19–21], the statistical properties and operating characteristics of the mutational profiling approach have yet to be well defined.

We investigated whether mutational profiling from whole exome sequencing can distinguish between clonal and independently arising tumors in SBBC and several other cancer types. The Clonal Likelihood Score (CLS) test statistic was computed as the percentage of high-confidence (HC) mutations shared by both tumors, out of the total number of HC mutations identified in the pair. A formal statistical test was developed and recommended parameters were defined using tumor pairs (mainly breast cancer) of known clonal status in The Cancer Genome Atlas (TCGA) database. We validated the test using recommended parameters on five independent datasets with known or putative clonal status from TCGA and the literature, including renal cell carcinoma, testicular cancer, and colorectal cancer. We then applied the CLS test to whole-exome sequencing data from two SBBC cases of unknown clonal status at our institution, and validated our conclusions with targeted deep sequencing. We also evaluated the biological evidence for our resulting call of metastatic SBBC. We developed recommendations for carrying out the CLS test on tumor mutational profiling data.

## Materials and Methods

### Sequencing methods

Tissue specimens were collected by the Moores Cancer Center Biorepository from consented patients under a University of California, San Diego Human Research Protections Program Institutional Review Board approved protocol (HRPP#050887 then 090401). Biorepository subjects provide a written consent which is maintained in the Biorepository archives. Detailed sequencing methods are in S1 Text. Whole exome sequencing and data processing. Briefly, an Illumina HiSeq 2000 was used to sequence whole exome DNA libraries captured with Agilent SureSelect at targeted coverage depth of 50x for germline and 200-400x for tumor DNA from fresh frozen specimens. Tumor purity, ploidy and absolute copy numbers were estimated using absCN-seq [22]. We validated a selected set of called mutations from each tumor pair using targeted sequencing to depth 8000x (MiSeq; Omegabiotek, Inc.). For validation data, variants were called using MuTect [23].

### Identification of high confidence (HC) mutations

We called single nucleotide variants (SNVs) in each tumor and germline sample using GATK [24] with default parameters, prioritizing specificity over sensitivity to detect a tumor mutation compared to matched germline DNA. We then applied additional stringent filtering rules to retain only high confidence (HC) somatic SNV’s; in germline DNA, we required at least 15 high-quality reads (Q>20) with at most one read supporting an alternate call, and also required < 5% alternate allele fraction. We filtered out tumor SNVs that were also present in the database of single nucleotide polymorphisms (dbsnp) 135 database [25], as these are likely germline variants. Finally, we retained as HC mutations those SNVs which had adequate coverage (40X) in both tumors at the affected locus; i.e. HC mutations were variants called at HC in at least one tumor, with adequate coverage in both tumors and with a high confidence homozygous reference call in germline DNA.

## Results

### The proposed statistical test using the Clonal Likelihood Score

Let *n* denote the total number of genomic loci where a mutation was called in either tumor, and let *X* denote the number of such loci where the mutation is shared. The CLS test is based on the proportion of shared mutations *p* out of *n* total mutations identified in a tumor pair, that is, *p* = *X/n*. Since *n* is known and can be considered fixed, *X* can be modeled as Binomial with parameters *n* and *p*.

A high rate of shared mutations is evidence in favor of clonal status, and so the CLS can be used to test the null hypothesis of tumor independence, H_0_: *p<p*_*0*_, against the alternative hypothesis Ha: *p>p*_0_. Here, *p*_*0*_ is the maximum rate of shared mutations expected to be called among independent tumor pairs, either from chance occurrence of true shared mutations, such as driver mutations, or from false positive (FP) calls from a given sequencing technology. In order for the CLS to have acceptable specificity and control of Type I error, *p*_*0*_ must be set at an appropriate empirically determined rate, as discussed below. The CLS test rejects the hypothesis of independence in favor of clonal status if CLS > *c/n*, where c is the critical value from an exact one-sided binomial test at the 5% significance level with parameters *n* and *p*_*0*_ (Table 1). We recommend reporting Clopper-Pearson exact two-sided confidence intervals [26] for the CLS, along with the observed p-value. The power of the CLS test to detect a truly clonal pair will depend on the total number of mutations *n*, and the expected proportion of shared mutations among clonal pairs, denoted as *p*_*a*_.

**Table 1:** The critical value for the CLS test (alpha = 0.05) and the number of total mutations needed to achieve 80% power.

p_0_ (Rate of shared HC mutations in independent tumor pairs)	p_a_ (Rate of shared HC mutations in clonal tumor pairs)	n (Total # of HC mutations needed to achieve 80% power)	c (Critical value for X in CLS test))
3%	10%	66	5
3%	15%	36	4
3%	20%	21	3
4%	10%	101	8
4%	15%	44	5
4%	20%	21	3

Table 1 Caption: Computations based on an exact binominal distribution under reasonable assumptions on *p*_*0*,_ the background rate of called shared mutations in independent tumor pairs, and *p*_*a*,_ the expected rate of called shared mutations in clonal tumor pairs. Under recommended assumptions, at least 44 called HC mutations are needed to implement the CLS test. The appropriate values of *p*_*0*_ and *p*_*a*_ will depend on the sequencing technology and tumor type and should be chosen prior to conducting the CLS test. The CLS test rejects the hypothesis of independence in favor of clonal status if CLS > c/n,

### Operating characteristics of the CLS test

The Type I error of the CLS test is governed by *p*_*0*_: if *p*_*0*_ is chosen to be lower than the actual rate of shared mutations in independent tumors, the test will reject the null hypothesis when tumors are actually independent, and the Type I error of the test will be greater than the nominal value of 5%. Also, it is clear that with too few total mutations in the pair of tumors, the CLS test will not have enough information to reliably distinguish clonal from independent tumors. We consider achieving 80% power to detect a truly clonal tumor pair as acceptable. Once *p*_*0*,_ is determined, the number of mutations *n* needed to achieve 80% power depends on *p*_*a*_, the rate of shared mutations in the clonal pair. We will determine the two critical parameters *p*_*0*_ and *p*_*a*_ by using empirical data from TCGA (see Table 1).

### Establishing parameters of the CLS using data from TCGA and the literature

To establish the parameters of the CLS, we computed the empirical distribution of the CLS from independent unrelated tumors in the TCGA (June 2012 release) [27]. We downloaded the called somatic mutations for all ER+ or triple negative breast tumors in TCGA with exome sequencing data. We found 357 ER+ and 46 TN tumors with median number of mutations 47 and 68 mutations per tumor, respectively (range 7–449). We paired each ER+ with each TN tumor to form 357 x 46 = 16,422 independent counterfactual tumor pairs and computed the CLS; of these, 98.4% (16,167) had CLS = 0. Among the 255 tumor pairs with a nonzero CLS, two pairs had two shared mutations and the remainder had one. The maximum observed CLS was 2.8%. As expected, the known driver genes PIK3CA (n = 122) and TP53 (n = 33) together accounted for 155 (60.3%) of the 257 total shared mutations. Thus, TCGA data show that of >16,000 independent breast cancer tumor pairs the maximum CLS was 2.8%. However, in two tumors from the same patient and sequenced in the same batch, it is expected that FP calls of a shared mutation due to technical artifacts would occur more often than in two different patients sequenced independently, and this would tend to increase *p*_*0*_ in patient testing. Hence we recommend allowing for a high background rate of false positive shared mutation calls in independent tumors, setting *p*_*0*_ = 3% to 4% (Table 1). Setting p_0_ higher than minimally necessary reduces the probability of Type I error at the cost of an increase in *n*, the number of total mutations needed, and empirically appears to leave *n* well within the realm of clinical sequencing efforts (see below). Thus, the proposed CLS test should be applicable to any variant calling assay which has a FP rate for shared mutations below 3%-4%.

We also investigated the distribution of the CLS in known clonal tumor pairs. In total we found fifteen primary and metastatic tumor pairs with genome wide mutation profiling data in TCGA (July 2015 release) across ten tumor types (S1 Table. TCGA Training Set). In these clonal pairs, the CLS values lie in the range of 14%-69% with a mean of 45%. We also identified one published study which profiled a metachronous primary and metastatic tumor breast cancer pair [28], with 48 shared of 50 total mutations called (CLS = 96%). Thus the available data indicate the rate of shared mutations in the vast majority of clonal tumor pairs is likely greater than 15%. Hence using *p*_*a*_ = 15% or 20% as a lower bound for the rate of shared mutations expected to occur in clonal tumors appears to conservatively allow for adequate power. The number of mutations needed for the CLS test to achieve 80% power under reasonable assumptions is given in Table 1. For example, given *p*_*0*_ = 4%, if a shared mutation rate of 15% or greater is expected in clonal tumors, at least 44 called HC mutations are needed to have 80% power to detect clonal status, using the CLS test. If we consider that a 20% rate of shared mutations can be expected in clonal tumors, then only 21 total mutations observed for the tumor pair are needed to achieve 80% power. Any variant calling method which attains sensitivity to detect shared mutations set by *p*_*a*_ would have adequate power for the CLS test.

### Validation of the CLS test in independent datasets with known clonal status

To investigate the performance of our CLS test, we assembled five validation datasets which had both known independent and known clonal tumor pairs, from TCGA and the published literature. Two sets of tumor pairs known to be clonal were assembled from a recent publication which studied inter-tumoral genomic variation in renal clear cell carcinoma [29]. An accompanying set of paired independent tumors in renal clear cell carcinoma was assembled from TCGA data, using the same random pairing approach as for the breast cancer tumors above. As a fourth data set, in TCGA, we also found five patients who each had two independent primary testicular tumors. Finally, a published study of synchronous primary and metastatic colorectal cancer included both putative independent and clonal synchronous tumors. In total, using three additional cancer types, we assembled 15,758 independent tumor pairs and 283 clonal pairs across these five data sets. We applied the recommended CLS test with *p*_*0*_ = 4% at 5% significance level to these data and report its performance.

We first report the independent tumor pairs from the validation data. 178 patients with primary renal clear cell carcinoma from TCGA were randomly paired to form 15,753 independent tumor pairs. Renal clear cell carcinoma was chosen because the same tumor type had available clonal pairs [29]. We also included five patients with two primary testicular germ cell tumors found in TCGA, representing more realistic independent pairs within the same patient (S2 Table. Independent Validation). For these 15758 independent pairs, the minimum p-value was 0.48 and the maximum CLS was 4.1%, consistent with expectations. Thus the CLS would correctly classify these tumor pairs as independent.

Considering the clonal tumor pairs, a recent study of intratumor heterogeneity sampled 5–11 biopsies per tumor for ten patients with renal clear cell carcinoma [29]. Within a patient, these biopsies are clonal by nature since they all come from the same bulk tumor. By randomly pairing biopsies within each patient, 237 within-tumor clonal pairs can be formed. For these pairs, the CLS values lie in the range of 13%-100% with a mean of 61% and the maximum p-value was <0.01 (S3 Table. Clonal Validation Tier 1). Thus the CLS would correctly classify these tumor pairs as clonal. In addition, four patients from the same publication had distant metastases. By randomly pairing the metastases with the related biopsies per patient, 46 primary-metastatic clonal pairs can be formed. The CLS values for these clonal pairs lie in the range of 23%-95% with a mean of 57% and a maximum p-value <10^−10^ (S4 Table. Clonal Validation Tier 2). Therefore, on this validation dataset, our proposed test could perfectly distinguish clonal tumors from independent tumors using a standard p-value cutoff of 0.05.

Next, we applied our test to the validation dataset that had the strongest similarity to our proposed setting of clinical testing for synchronous tumors. Lee et al. characterized fifteen pairs of synchronous primary and metastatic colorectal tumors at the time of diagnosis by both SNP array and exome sequencing [30]. Using unsupervised hierarchical clustering of somatic copy number alterations (SCNA) inferred from the SNP array data, they concluded that eight pairs clustered together and thus may be considered closely related (considered as equivalent to clonal origin in our case), and seven pairs did not cluster together and thus were remotely related (considered equivalent to independent origin in our case). We compared our CLS test results based on the exome sequencing data to their results based on the SCNA data (S5 Table. Clonality Calls: Somatic Copy Number Alterations vs CLS in Synchronous Primary and Metastatic Colorectal Tumors). By using a standard p-value cutoff of 0.05, two clearly separated classes were obtained by our test. For the assumed clonal cases, the CLS values lie in the range of 32%-54% with a mean of 41%. For the assumed independent cases, the maximum CLS value is 4.1%, which is highly consistent with the TCGA exploratory results and *p*_*0*_ parameter we set. We had only one discordant case with Lee’s result. Patient 353 was identified as remotely related (independent origin) by the clustering analysis of SCNAs, however the tumor pair had 31 shared mutations out of 59 total mutations with a CLS of 53%, making it extremely unlikely to be of independent origin. Lee et al. noticed that patient 353 had the fewest number of SCNA (in particular loss of heterozygosity) among all the patients [30]. However, an issue with this approach is that testing tumors for clonal status based on SCNA is known to be problematic for nearly diploid genomes [11]. Furthermore, such a clonal status test employing hierarchical clustering has been shown to be suboptimal [18]. Therefore, most likely this discordant case does not suggest that our test made an erroneous call. Instead, it seems to represent a good example demonstrating the advantage of an SNV based test over the SCNA based test. Taken together, our CLS test has been well validated using these independent datasets. We then generated sequencing data for our SBBC samples and applied the test to them.

### Application of the CLS test to two cases of SBBC of unknown clonal status

#### Patient and sample characteristics

We obtained germline DNA and fresh frozen tumor samples from two women who presented with synchronous bilateral node-negative invasive breast cancers, each with one tumor ER+/PR+/HER2- (HR+) lobular and the other triple negative (TN) ductal (Table 2). Patient 1 was an African American woman age 75 years with an HR+ infiltrating lobular carcinoma, stage 2, grade 1 (ID: PT1HR+), and a TN invasive ductal breast cancer, stage 2, grade 2 (ID: PT1TN) in the contralateral breast. Patient 2 was a 70 year old non-Hispanic white woman with an HR+ infiltrating lobular carcinoma, stage 1c, grade 1 (ID: PT2HR+); her contralateral tumor was TN invasive ductal carcinoma, stage 1c, grade 3 (ID: PT2TN). With 73 and 44 months of follow up, respectively, neither patient has recurred.

**Table 2:** Tumor sample characteristics for two cases of Synchronous Bilateral Breast Cancer (SBBC).

Patient	Sample ID	Histology	Phenotype	Cellularity (Estimated by pathologist)	Cellularity (Estimated by absCNseq)	Ploidy (Estimated by absCNseq)
Patient 1	PT1TN	Invasive ductal carcinoma (apocrine ca)	TN	~70%	47%	3.43
Patient 1	PT1HR+	Invasive lobular carcinoma (pleomorphic)	ER+/HER2-	~40%	31%	3.06
Patient 2	PT2TN	Invasive ductal carcinoma	TN	~70%	77%	1.88
Patient 2	PT2HR+	Invasive lobular carcinoma	ER+/HER2-	~70%	56%	2.34

#### Exome sequencing and application of the CLS test

We identified 50 to 100 HC mutations per tumor (Table 3). For patient 1, 50 and 62 HC somatic mutations were called in the HR+ and TN tumors, respectively. All 50 of the HC mutations identified in the HR+ tumor were shared by the TN tumor, for a CLS of 100 X 50/62 = 81%. For patient 2, 81 and 105 HC SNV candidates, were identified in the HR+ and TN tumors respectively, of which two were shared, for a CLS of 100 X 2 / 184 = 1.1%.

**Table 3:** Number of shared and private high confidence (HC) somatic mutations and Clonal Likelihood Score in two SBBC tumor pairs, identified from exome sequencing and stringent filtering.

	Private HC mutations:TN specific	Private HC mutations: HR+ specific	Shared HC mutations	Total HC mutations in tumor pair	Clonal Likelihood Score (CLS) (Before validation)	Clonal Likelihood Score (CLS) (After validation)
Patient 1	12	0	50	62	81% (50/62)	100% (51/51)
Patient 2	103	79	2	184	1% (2/184)	0% (0/182)

For patient 1, an exact binomial test of *H*_*0*_: *p<0*.*04* against a one sided alternative, with *X* = 50 successes in *n* = 62 trials, yields *p*<1 x 10^−15^, rejecting the null hypothesis of independence. An exact Clopper-Pearson 95% confidence interval for the CLS is 69% to 90%. This establishes strong evidence in favor of clonal status for patient 1.

For patient 2, an exact binomial test of H_0_: *p*<0.04 against a one sided alternative, with *X* = 2 successes in *n* = 184 trials, yields *p* >0.99, failing to reject the hypothesis of independence. The 95% Clopper-Pearson confidence interval is 0.1% to 4%, consistent with independence, and well below shared mutation rates observed in TCGA.

#### Independent validation of called HC mutations

**Fig 1:**
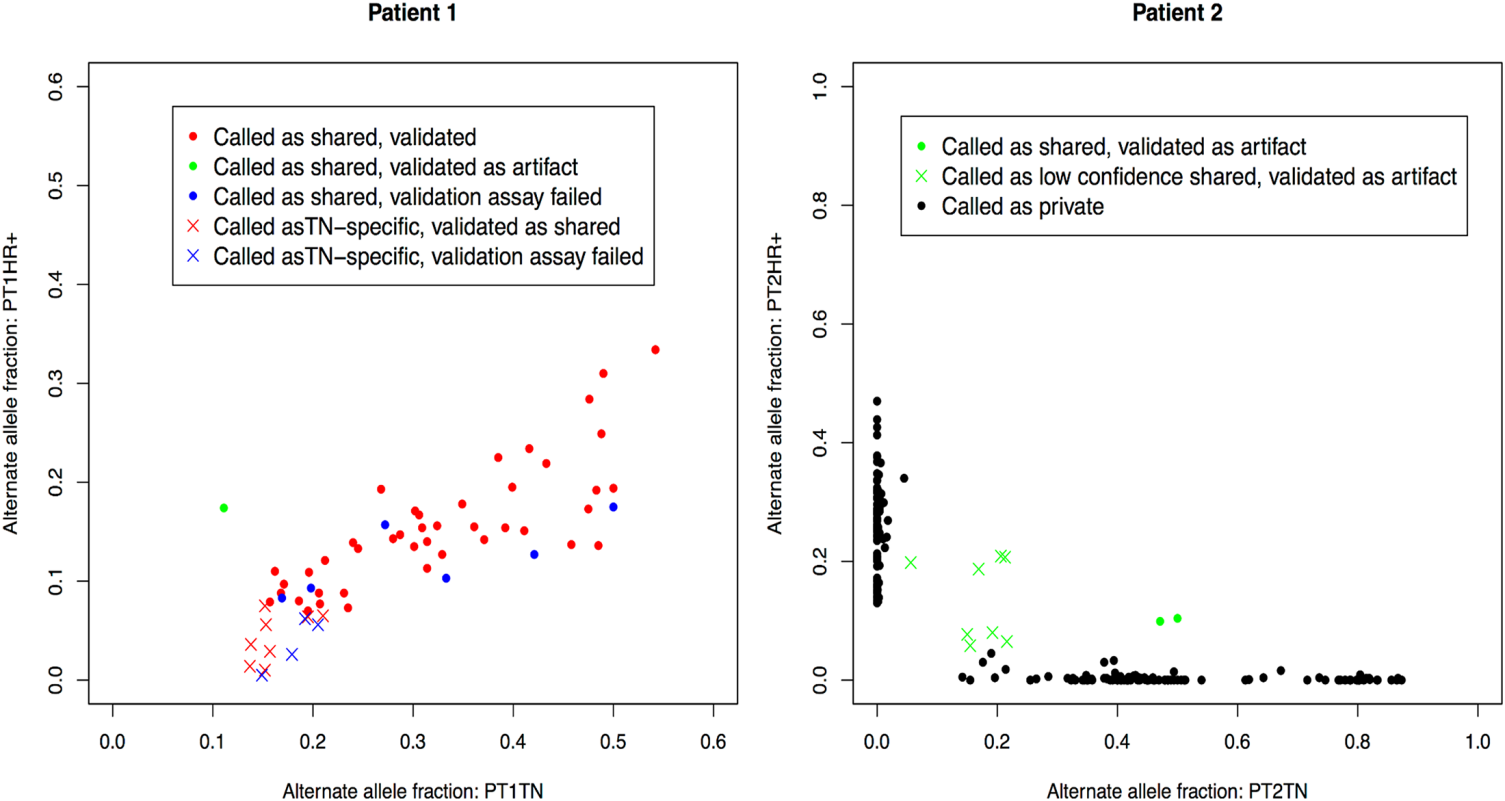
Distinct patterns of mutant allelic fraction for two tumor pairs. Left panel, Patient 1, clonal; right panel, Patient 2, independent. Each dot represents a HC shared mutation identified from exome sequencing and stringent filtering, with the proportion of alternatively called alleles plotted on the y-axis for the HR+ tumor and on the x-axis for the TN tumor. The x’s represent additional loci sent for resequencing in the validation assay to confirm negative calls for shared mutations. Y-axis: HR+ tumor; x-axis: TN tumor. Red dots: HC shared mutations, confirmed by the targeted deep sequencing validation assay. Red crosses: mutations called as HC private mutations, validated as true shared mutations. Green dots: putative HC mutations determined to be false positives by the validation assay. Blue color: the validation assay failed in at least one tissue (germline or 2 tumors). Black dots: private HC somatic mutations not sent for validation. Green crosses: low-confidence potential SNV’s selected for validation (>5% prevalence in both tumors), all confirmed to be sequencing artifacts (no alternate allele detected in either germline or tumor DNA by validation assay). For both patients, the false positive calls (green dots) are observed to be separate from the other called mutations. For patient 1, where all validated mutations were confirmed as shared, the false negative calls for shared mutations are seen to be shared mutations present at low allelic fraction (Fig 1 left, red crosses). For patient 2, all validated mutations were called as private. The confirmed negative loci (Fig 1 right, green crosses) were all low-confidence possible SNV’s which validated as germline homozygous reference. The distinct patterns seen in the figures again suggest that the tumors from patient 1 were clonal and while those from patient 2 arose independently.

We used an independent targeted deep sequencing assay to validate the called shared mutations. Fig 1 shows the allelic fraction of validated, false positive, and false negative sequencing calls for each patient’s tumor pair. The observed patterns are seen to be distinct. For patient 1, all 50 identified HC shared mutations (Table 3) were resequenced, and six of them failed the assay in at least one of the three DNA samples. Of 44 sites remaining, 43 were confirmed as shared. One site validated as a false positive call and was seen to be homozygous reference in both germline and tumors. The 12 identified HC private mutations (Table 3) were also re-sequenced, with four assay failures and eight confirmed as true shared mutations and thus false private calls. The observed mutant allelic fractions for the eight false negative calls in the HR+ tumor whole-exome sequencing data were all below 7.5%, explaining why they were initially missed. Thus for patient 1, among the 52 putative mutations for which we obtained validation data, 51 were in fact shared and one was a false positive call, consistent with a clonal origin. The validated CLS was 100 x 51/51 = 100%.

For patient 2, we re-sequenced the two called HC shared mutations. We also re-sequenced all loci (none were called as HC variants) with an alternate allele prevalence > 5% in both tumors (n = 8), as these appeared to be the most likely candidates for a false negative call. All ten mutations sent were confirmed as sequencing artifacts, with homozygous reference genotype in the germline and two tumor DNA samples. Thus, there were no confirmed shared mutations out of 184 HC candidates, consistent with independence. The estimated false positive rate for this putative independent pair was about 1% (2/184 = 0.011). The validated CLS was 100 x 0/182 = 0%.

In our own data, the FP rate for shared mutations was about 1% of total called mutations in the putative independent SBBC pair; detailed analysis of false positive and false negative rates from targeted deep sequencing is in S1 Text, Whole exome sequencing and data processing.

#### Clonal evolution and shared actionable mutations in the clonal SBBC tumor pair

**Fig 2:**
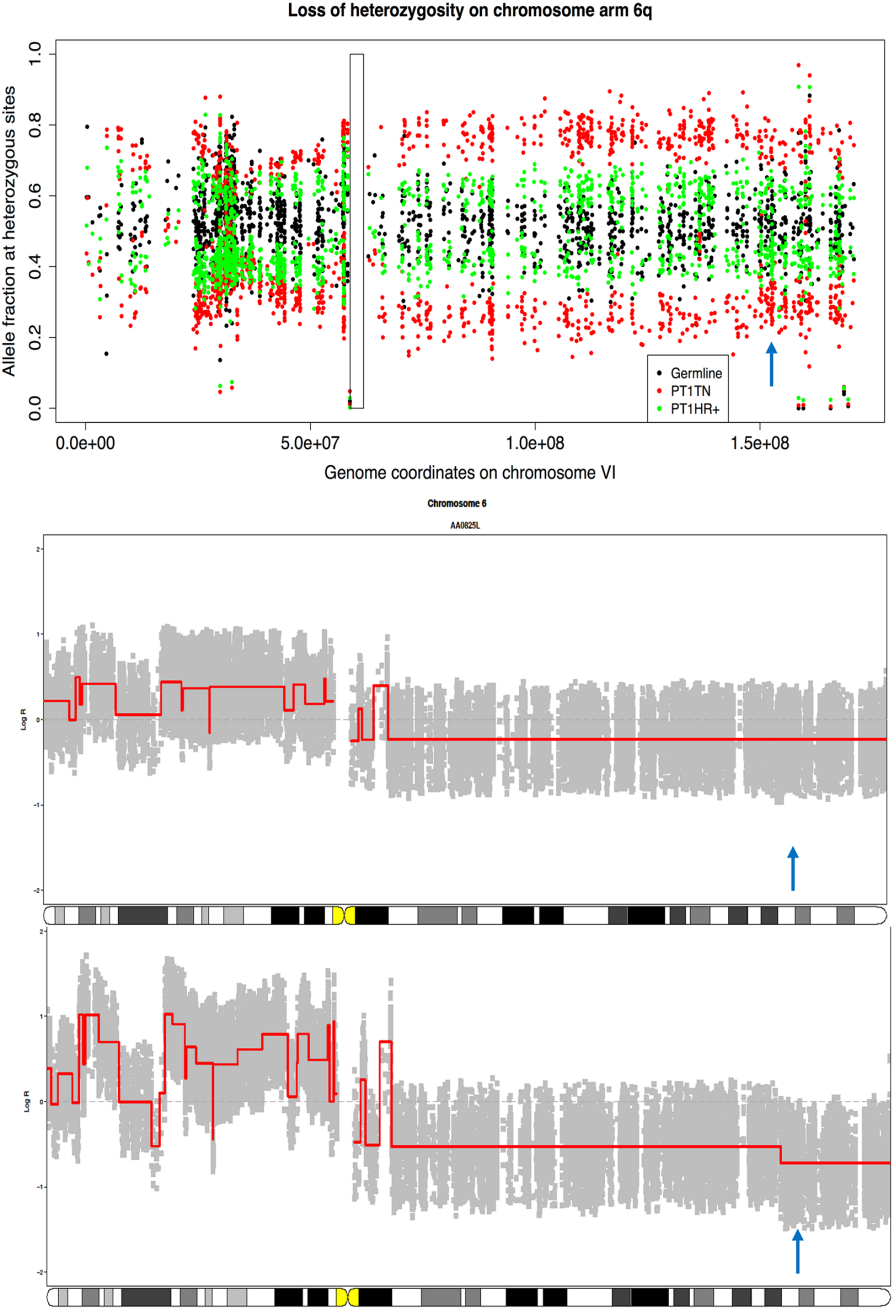
Copy number alterations potentially changing the ER status of the clonal tumors from Patient 1. Upper panel: Loss of heterozygosity (LOH) profiles on chromosome arm 6q. Two separated bands of allele fractions along the chromosome, a typical consequence of LOH, is clearly seen in TN tumor (red dots) but is less evident in HR+ tumor (green dots) due to low cellularity of the sample. Rectangle delimits the centromere and the blue arrow points to the ER locus. Middle and lower panel: segmented log copy ratio profiles on chromosome 6 for the HR+ and TN tumor respectively, produced by copy number package [33]. One copy loss of distal end of 6q encapsulating the ER locus (blue arrow) is seen in the TN tumor but not in HR+ tumor. A plausible evolutionary sequence is an initial copy neutral LOH affecting the entire 6q chromosome arm in the primary tumor followed by a new deletion at the ESR1 locus, which reduced ER expression in the metastatic TN tumor.

We investigated possible mechanisms for the change from ER+ to ER- in the clonal tumor pair. We examined copy number alterations on chromosome 6q where *ESR1* is located and observed large-scale loss-of-heterozygosity (LOH) common to both tumors (Fig 2, upper panel). There was an additional deletion in the distal end of 6q (6q25.1-6q27) apparent only in the TN tumor (Fig 2, lower panel), covering the entire *ESR1* locus. We then applied absCN-seq to estimate the absolute copy numbers for the *ESR1* region (Table 1) and found that the HR+ tumor has two copies and the TN tumor only one copy for this region. Thus a plausible evolutionary sequence is that a copy neutral LOH affected the entire 6q chromosome arm in the primary tumor. Copy neutral LOH is known to be associated with gene expression changes [31], but in our case did not seem to ablate ER expression. However, during migration to the contra-lateral breast, a new deletion was established in the TN tumor at the ESR1 locus, either from subclone expansion or from a de novo mutation, and this rendered ER expression undetectable. Interestingly, this deletion also strikes ARID1B, a new tumor suppressor gene found in breast cancer [32].

In addition, we identified several known shared driver alterations including chromosome 8 amplifications affecting *FGFR1*, *ZNF703* and *MYC*, as well as a nonsense mutation (L298*) in gene *NF1* and a deleterious missense mutation (E1436A) in gene *NCOR1* [32]. Among these, FGFR1, *MYC* and *NF1* alterations are potentially actionable [34, 35], demonstrating the potentially important therapeutic implications of profiling SBBC for clonal origin.

## Discussion

We have developed a carefully defined and effective approach using mutational profiling from next generation sequencing data to distinguish between clonal and independently arising tumors in SBBC, and potentially in other cancer types. The Clonal Likelihood Score (CLS) is computed as the percentage of High Confidence mutations which are shared by both tumors, out of the total number of distinct genomic loci with High Confidence mutations identified in the pair. The CLS test is implemented as a one sample binomial test of proportions, with null hypothesis of independence, and recommended test parameters determined empirically from TCGA data. Importantly, the data suggest that up to 2% of tumors from different patients may have at least one shared mutation, arising from common driver mutations, technical artifacts, and shared passenger mutations arising by chance, and the rate is higher, up to 4%, for tumors arising within the same patient. Thus, careful implementation of the CLS test such as we propose here is needed to control error rates. Using data from TCGA and the literature, we have identified conditions under which the CLS test can be expected to have at most 5% Type I error rate, (falsely identifying an independent pair as clonal), and also adequate power (80%) to correctly detect a clonal tumor pair, using high confidence somatic mutation calls.

The CLS test has a null hypothesis of CLS ≤ 4% against a one-sided alternative; the test achieves 80% power to detect a CLS of 15% if there are at least 44 HC mutations called in the tumor pair. We validated the CLS test in five independent data sets containing both clonal and independent tumors, with various tumor types: renal cell carcinoma, testicular cancer and colorectal cancer. In these validation data, the CLS test correctly called all but one of 15,758 putative independent tumor pairs as independent, with maximum CLS of 4.1% and minimum p-value of 0.48. We believe the one discordant result (CLS = 0.52) was likely a clonal pair which was missed due to low power of the copy number based analysis which was used to classify this pair as independent in the published paper from which we drew these data. The CLS test also correctly called all 283 clonal pairs in the validation data as clonal, with minimum CLS 13% (mean 61%, maximum 100%) and maximum p-value p< 0.01.

We then applied the formal CLS test to exome-wide sequencing data on two cases of invasive SBBC of unknown clonal status, each with one HR+ lobular and TN ductal carcinoma. In one SBBC patient, the CLS was 81% (95% CI: 69%, 90%) shared out of 62 total HC mutations called (p< 0.0001), establishing clonal origin of the two tumors. In the other patient, the CLS was 1.1% (95% CI: 0.1%, 4%) shared of 184 total HC mutations called (p = 0.99), consistent with independent origin. For the clonal SBBC case, we were able to determine the likely primary tumor by investigation of copy number alterations and to identify a plausible mechanism of the change from ER+ to ER- during metastasis. We also discovered a novel potential driver alteration (e.g. 6q25.1-6q27 deletion) associated with metastasis but not needed to adapt to a new physiological environment outside the breast, highlighting the unique biological information available in SBBC. It is also important to note that two clinical measures currently used to identify clonal SBBC are concordant histological features and presence of systemic metastases [6]; however, our results show these features are not necessarily present in clonal tumors. In the future, improved assessment of clonal or independent status may support better estimates of prognosis in SBBC. Furthermore, it is possible that the association of SBBC with increased familial risk may not apply to patients with clonal tumors, which might result in different patient management. These observations highlight the potential clinical importance of our results.

For adequate control of Type I error in the CLS test, the mutational profiling pipeline should maintain a call rate of shared mutations of 4% or less in independent tumors. To achieve this, we recommend using a moderately sensitive, but highly specific, variant caller and stringent filtering rules. We used GATK with default parameters and required adequate information across all three tissues at the same locus (germline and two tumor samples). We used targeted deep sequencing (8000x) to validate our results, and our validation data support the use of this approach (S1 Text. Whole exome sequencing and data processing).

To obtain adequate power, a true shared mutation must have an adequate chance to be detected in both tumor samples, and there must be at least a total of 44 mutations called considering both tumors, under our recommended test parameters. In our data, the major source of false negative errors for detecting a shared mutation was from low cellularity in one tumor of a pair, with the consequence that some shared mutations were falsely called as private mutations. In a given mutational profiling pipeline, lower detection rates for true shared mutations can be compensated for in the CLS test by lowering the alternative hypothesis rate *p*_*a*_, for example lowering *p*_*a*_ from 15% to 10%. This maintains 80% power to detect clonal status, while increasing the total number of mutations required from 44 to 101 as seen in Table 1.

In conclusion, we have developed the statistical properties of a well-defined Clonal Likelihood Score test based on mutational profiling of tumor DNA. We developed the test using TCGA tumor data, and validated the test in five additional sets of tumors from TCGA and the literature, comprising four cancer types. We then applied the test to two cases of SBBC at our own institution, and obtained results with potential biological interest and clinical relevance. Under reasonable conditions, the CLS test appears to reliably distinguish between tumors of clonal and of independent origin. This approach may have applicability to clinical sequencing efforts in synchronous bilateral breast cancer and in other cancer types, and may have scientific and clinical utility.

## Supporting Information

S1 Table TCGA Training Set. (XLSX)

S2 Table Independent Validation. (XLSX)

S3 Table Clonal Validation Tier. 1. (XLSX)

S4 Table Clonal Validation Tier 2. (XLSX)

S5 Table Clonality Calls: Somatic Copy Number Alterations vs CLS in Synchronous Primary and Metastatic Colorectal Tumors. (DOCX)

S1 Text Whole exome sequencing and data processing. (DOCX)

## References

[1] Schmid SM, Pfefferkorn C, Myrick ME, Viehl CT, Obermann E, Schotzau A, et al Prognosis of early-stage synchronous bilateral invasive breast cancer. Eur J Surg Oncol. 2011;37(7):623–8. 10.1016/j.ejso.2011.05.006 21628090

[2] Brennan ME, Houssami N, Lord S, Macaskill P, Irwig L, Dixon JM, et al Magnetic resonance imaging screening of the contralateral breast in women with newly diagnosed breast cancer: systematic review and meta-analysis of incremental cancer detection and impact on surgical management. J Clin Oncol. 2009;27(33):5640–9. 10.1200/JCO.2008.21.5756 19805685

[3] Banelli B, Casciano I, Di Vinci A, Gatteschi B, Levaggi A, Carli F, et al Pathological and molecular characteristics distinguishing contralateral metastatic from new primary breast cancer. Ann Oncology. 2010;21(6):1237–42. 10.1093/annonc/mdp470 19875753

[4] Saad RS, Denning KL, Finkelstein SD, Liu Y, Pereira TC, Lin X, et al Diagnostic and prognostic utility of molecular markers in synchronous bilateral breast carcinoma. Mod Pathol. 2008;21(10):1200–7. 10.1038/modpathol.2008.35 18469799

[5] Nichol AM, Yerushalmi R, Tyldesley S, Lesperance M, Bajdik CD, Speers C, et al A case-match study comparing unilateral with synchronous bilateral breast cancer outcomes. J Clin Oncol. 2011;29(36):4763–8. 10.1200/JCO.2011.35.0165 22105824

[6] Leis HP Jr. Bilateral breast cancer. Surg Clin North Am. 1978;58(4):833–41. 356301 10.1016/s0039-6109(16)41595-1

[7] Imyanitov EN, Suspitsin EN, Grigoriev MY, Togo AV, Kuligina E, Belogubova EV, et al Concordance of allelic imbalance profiles in synchronous and metachronous bilateral breast carcinomas. Internat J Cancer. 2002;100(5):557–64. 10.1002/ijc.10530 12124805

[8] Teixeira MR, Ribeiro FR, Torres L, Pandis N, Andersen JA, Lothe RA, et al Assessment of clonal relationships in ipsilateral and bilateral multiple breast carcinomas by comparative genomic hybridisation and hierarchical clustering analysis. Br J Cancer. 2004;91(4):775–82. 15266323 10.1038/sj.bjc.6602021 PMC2364777

[9] Janschek E, Kandioler-Eckersberger D, Ludwig C, Kappel S, Wolf B, Taucher S, et al Contralateral breast cancer: molecular differentiation between metastasis and second primary cancer. Breast Cancer Res Treat. 2001;67(1):1–8. 11518461 10.1023/a:1010661514306

[10] Ostrovnaya I, Seshan VE, Begg CB. Comparison of Properties of Tests for Assessing Tumor Clonality. Biometrics. 2008;64(4):1018–22. 10.1111/j.1541-0420.2008.00988.x 18266893 PMC2761024

[11] Ostrovnaya I, Olshen AB, Seshan VE, Orlow I, Albertson DG, Begg CB. A metastasis or a second independent cancer? Evaluating the clonal origin of tumors using array copy number data. Stat Med. 2010;29(15):1608–21. 10.1002/sim.3866 20205270 PMC3145177

[12] Andrade VP, Ostrovnaya I, Seshan VE, Morrogh M, Giri D, Olvera N, et al Clonal relatedness between lobular carcinoma in situ and synchronous malignant lesions. Breast Cancer Res. 2012;14(4):R103 10.1186/bcr3222 22776144 PMC3680923

[13] Begg CB, Eng KH, Hummer AJ. Statistical tests for clonality. Biometrics. 2007;63(2):522–30. 17688504 10.1111/j.1541-0420.2006.00681.x PMC2736112

[14] Chunder N, Roy A, Roychoudhury S, Panda CK. Molecular study of clonality in multifocal and bilateral breast tumors. Pathol Res Pract. 2004;200(10):735–41. 15648612 10.1016/j.prp.2004.09.001

[15] Heselmeyer-Haddad K, Garcia LYB, Bradley A, Ortiz-Melendez C, Lee WJ, Christensen R, et al Single-Cell Genetic Analysis of Ductal Carcinoma in Situ and Invasive Breast Cancer Reveals Enormous Tumor Heterogeneity yet Conserved Genomic Imbalances and Gain of MYC during Progression. Am J Pathol. 2012;181(5):1807–22. 10.1016/j.ajpath.2012.07.012 23062488 PMC3483801

[16] Ostrovnaya I, Seshan VE, Olshen AB, Begg CB. Clonality: an R package for testing clonal relatedness of two tumors from the same patient based on their genomic profiles. Bioinformatics. 2011;27(12):1698–9. 10.1093/bioinformatics/btr267 21546399 PMC3106202

[17] Wagner PL, Kitabayashi N, Chen YT, Shin SJ. Clonal relationship between closely approximated low-grade ductal and lobular lesions in the breast: a molecular study of 10 cases. Am J Clin Pathol. 2009;132(6):871–6. 10.1309/AJCP7AK1VWFNMCSW 19926578

[18] Ostrovnaya I, Begg CB. Testing clonal relatedness of tumors using array comparative genomic hybridization: a statistical challenge. Clinical Cancer Research. 2010;16(5):1358–67. 10.1158/1078-0432.CCR-09-2398 20179213 PMC2831112

[19] De Mattos-Arruda L, Bidard FC, Won HLH, Cortes J, Ng CKY, Peg V, et al Establishing the origin of metastatic deposits in the setting of multiple primary malignancies: The role of massively parallel sequencing. Mol Oncol. 2014;8(1):150–8. 10.1016/j.molonc.2013.10.006 24220311 PMC5528499

[20] Nemes S, Danielsson A, Parris TZ, Jonasson JM, Bulow E, Karlsson P, et al A diagnostic algorithm to identify paired tumors with clonal origin. Genes Chromosomes Cancer. 2013;52(11):1007–16. 10.1002/gcc.22096 23999905

[21] Poplawski AB, Jankowski M, Erickson SW, de Stahl TD, Partridge EC, Crasto C, et al Frequent genetic differences between matched primary and metastatic breast cancer provide an approach to identification of biomarkers for disease progression. Eur J Hum Genet. 2010;18(5):560–8. 10.1038/ejhg.2009.230 20051991 PMC2987320

[22] Bao L, Pu M, Messer K. AbsCN-seq: a statistical method to estimate tumor purity, ploidy and absolute copy numbers from next-generation sequencing data. Bioinformatics. 2014. Epub 2014/01/07. 10.1093/bioinformatics/btt759 PMC6280749 24389661

[23] Cibulskis K, Lawrence MS, Carter SL, Sivachenko A, Jaffe D, Sougnez C, et al Sensitive detection of somatic point mutations in impure and heterogeneous cancer samples. Nat Biotechnol. 2013;31(3):213–9. 10.1038/nbt.2514 23396013 PMC3833702

[24] DePristo MA, Banks E, Poplin R, Garimella KV, Maguire JR, Hartl C, et al A framework for variation discovery and genotyping using next-generation DNA sequencing data. Nat Genet. 2011;43(5):491–8. 10.1038/ng.806 21478889 PMC3083463

[25] Sherry ST, Ward MH, Kholodov M, Baker J, Phan L, Smigielski EM, et al dbSNP: the NCBI database of genetic variation. Nucleic Acids Res. 2001;29(1):308–11. 11125122 10.1093/nar/29.1.308 PMC29783

[26] Agresti A. Categorical Data Analysis: Wiley; 2013.

[27] Weinstein JN, Collisson EA, Mills GB, Shaw KR, Ozenberger BA, Ellrott K, et al The Cancer Genome Atlas Pan-Cancer analysis project. Nat Genet. 45(10):1113–20. 10.1038/ng.2764 24071849 PMC3919969

[28] Hernandez L, Wilkerson PM, Lambros MB, Campion-Flora A, Rodrigues DN, Gauthier A, et al Genomic and mutational profiling of ductal carcinomas in situ and matched adjacent invasive breast cancers reveals intra-tumour genetic heterogeneity and clonal selection. J Pathol. 2012;227(1):42–52. 10.1002/path.3990 22252965 PMC4975517

[29] Gerlinger M, Horswell S, Larkin J, Rowan AJ, Salm MP, Varela I, et al Genomic architecture and evolution of clear cell renal cell carcinomas defined by multiregion sequencing. Nat Genet. 2014;46(3):225–33. 10.1038/ng.2891 24487277 PMC4636053

[30] Lee SY, Haq F, Kim D, Jun C, Jo HJ, Ahn SM, et al Comparative genomic analysis of primary and synchronous metastatic colorectal cancers. PLoS One. 2014;9(3):e90459 10.1371/journal.pone.0090459 24599305 PMC3944022

[31] Hu N, Clifford RJ, Yang HH, Wang C, Goldstein AM, Ding T, et al Genome wide analysis of DNA copy number neutral loss of heterozygosity (CNNLOH) and its relation to gene expression in esophageal squamous cell carcinoma. BMC Genomics. 2010;11:576 10.1186/1471-2164-11-576 20955586 PMC3091724

[32] Stephens PJ, Tarpey PS, Davies H, Van Loo P, Greenman C, Wedge DC, et al The landscape of cancer genes and mutational processes in breast cancer. Nature. 2012;486(7403):400–4. 10.1038/nature11017 22722201 PMC3428862

[33] Nilsen G, Liestol K, Van Loo P, Moen Vollan HK, Eide MB, Rueda OM, et al Copynumber: Efficient algorithms for single- and multi-track copy number segmentation. BMC Genomics. 2012;13:591 10.1186/1471-2164-13-591 23442169 PMC3582591

[34] Balko JM, Giltnane JM, Wang K, Schwarz LJ, Young CD, Cook RS, et al Molecular profiling of the residual disease of triple-negative breast cancers after neoadjuvant chemotherapy identifies actionable therapeutic targets. Cancer Discov. 2014;4(2):232–45. 10.1158/2159-8290.CD-13-0286 24356096 PMC3946308

[35] Soria JC, DeBraud F, Bahleda R, Adamo B, Andre F, Dienstmann R, et al Phase I/IIa study evaluating the safety, efficacy, pharmacokinetics, and pharmacodynamics of lucitanib in advanced solid tumors. Ann Oncol. 2014;25(11):2244–51. 10.1093/annonc/mdu390 25193991

