# Supplementary material for: Mutational Profiling Can Establish Clonal or Independent Origin in Synchronous Bilateral Breast and Other Tumors
Source: PLoS One. 2015 Nov 10;10(11):e0142487. doi: 10.1371/journal.pone.0142487 (PMC4640562; doi:10.1371/journal.pone.0142487)
Supplement: S5 Table — (DOCX) [file pone.0142487.s005.docx]

| Clonality Calls: Somatic Copy Number Alterations vs CLS in Synchronous Primary and Metastatic Colorectal Tumors | | | | | | | | | |
| --- | --- | --- | --- | --- | --- | --- | --- | --- | --- |
|  | Tumor Regions | | Total Number of Mutations | | Number of Mutation Types | |  |  | Calls by Lee et al. |
| Set | Primary | Metastasis | Primary | Metastasis | Common | Unique | CLS | P |  |
| 1 | 278T | 278LT | 56 | 50 | 37 | 69 | 53.6 | 2.50E-33 | Closely related |
| 2 | 353T | 353LT | 41 | 49 | 31 | 59 | 52.5 | 8.44E-28 | Remotely related |
| 3 | 627T | 627LT | 30 | 26 | 18 | 38 | 47.4 | 1.07E-15 | Closely related |
| 4 | 413T | 413LT | 41 | 48 | 26 | 63 | 41.3 | 3.77E-20 | Closely related |
| 5 | 718T | 718LT | 37 | 49 | 24 | 62 | 38.7 | 6.18E-18 | Closely related |
| 6 | 523T | 523LT | 69 | 75 | 38 | 106 | 35.8 | 4.48E-26 | Closely related |
| 7 | 503T | 503LT | 48 | 52 | 26 | 74 | 35.1 | 4.53E-18 | Closely related |
| 8 | 707T | 707LT | 57 | 60 | 30 | 87 | 34.5 | 2.39E-20 | Closely related |
| 9 | 509T | 509LT | 55 | 76 | 32 | 99 | 32.3 | 1.27E-20 | Closely related |
| 10 | 381T | 381LT | 13 | 64 | 3 | 74 | 4.1 | 5.72E-01 | Remotely related |
| 11 | 262T | 262LT | 70 | 528 | 1 | 597 | 0.2 | 1.00E+00 | Remotely related |
| 12 | 721T | 721LT | 75 | 455 | 1 | 529 | 0.2 | 1.00E+00 | Remotely related |
| 13 | 185T | 185LT | 80 | 25 | 0 | 105 | 0 | 1.00E+00 | Remotely related |
| 14 | 250T | 250LT | 75 | 511 | 0 | 586 | 0 | 1.00E+00 | Remotely related |
| 15 | 526T | 526LT | 35 | 553 | 0 | 588 | 0 | 1.00E+00 | Remotely related |
