# Supplementary material for: Mutational Profiling Can Establish Clonal or Independent Origin in Synchronous Bilateral Breast and Other Tumors
Source: PLoS One. 2015 Nov 10;10(11):e0142487. doi: 10.1371/journal.pone.0142487 (PMC4640562; doi:10.1371/journal.pone.0142487)
Supplement: S1 Text — (DOCX) [file pone.0142487.s006.docx]

**S1 Text: Whole exome sequencing and data processing**

Agilent SureSelect All Exon 50Mb Target Enrichment Kits were used for exome capture. Following addition of unique DNA barcodes, samples for each patient were pooled (germline, tumor 1, tumor 2) and 200 base pair reads were sequenced by paired-end sequencing on the Illumina HiSeq 2000. Read depth gave a median base coverage at 50-60X for germline, at 240X for the three tumors with 70% cellularity and at 430X for the tumor with 40% cellularity. Raw sequencing data have been deposited in SRA with an accession number of SRP041629. Reads were aligned to the human reference genome (b37) using BWA aln (version 0.6.1) [1]. PCR duplicates were removed by PICARD (version 1.64). To identify SNV’s in the exome of each DNA sample, we used the genotype caller in GATK (version 1.5) pipeline [2] with default parameters after indel realignment, base quality recalibration and the recommended filtration. For copy number alteration detection and segmentation, we used VarScan2 [3], DNAcopy [4] and copynumber [5]. Tumor purity, ploidy and absolute copy numbers were estimated using absCN-seq [6]. Functional impacts of SNV’s were annotated using snpEff (version 2.0.5d) [7].

## Targeted sequencing of selected mutations as validation

We validated a set of called somatic mutations from each tumor pair using targeted deep sequencing (Omegabiotek, Inc.). PCR primers were designed using the Primer3 algorithm to amplify a region of ~200 bp surrounding the mutation to be validated. PCR reactions were carried out using the KAPA HiFi HotStart PCR kit (Kapa Biosystems, Wilmington, MA) following manufacturer's recommendations. An aliquot of each PCR reaction was run on a 2% agarose gel to verify the correct size of the amplicons. All PCR reactions from the same template DNA were pooled at a roughly equal ratio, and the amplicons further purified using the E.Z.N.A Cycle Pure kit (Omega Biotek, Norcross, GA). To generate Illumina-compatible NGS libraries, ~500 ng of the purified amplicon pools were barcoded using the TruSeq DNA Sample Prep Kit (Illumina, San Diego, CA). Six new libraries were multiplexed for 2x150 bp paired-end run on a MiSeq sequencer using the MiSeq Reagent Kit v2 (Illumina). Reads were aligned to the h19 genome using the MiSeq default alignment pipeline. Coverage of depth for loci of interest was computed using samtools [8]. We then used the MuTect software [9] to call somatic mutations from the targeted sequencing data.

## False positives and false negatives for calling shared mutations from our validation study

Our validation study also enables us to investigate the major sources of false positive (FP) and false negative (FN) calls for shared mutations. Importantly, the FP and FN here consider both tumors together in the light of a formal test introduced in the main text, and summarize the consolidated effects of different combination of individual erroneous calls arising from each tumor.

FP calls of a shared mutation might give erroneous evidence in favor of clonality, causing a Type I error. A FP arises when a shared mutation is called but in fact it is either a private mutation or there is no mutation at all. It should be very rare that a true private mutation in one tumor is misidentified by chance in the other tumor, and we did not observe any such FPs in our data. On the other hand, two scenarios can result in FPs when no somatic mutation exists at all (i.e. both tumors have the same genotype as the germline DNA). First, a FP occurs when a germline heterozygous genotype is erroneously called as homozygous reference in germline DNA but correctly called in both tumors. Our filtering rules are designed to minimize this possibility, and we did not see any such FP calls in our data. A second source of FP calls occurs when the homozygous reference genotype is somehow erroneously called heterozygous in both tumors, resulting most likely from sequencing and alignment artifacts that preferentially produce false SNV calls at a locus in both tumors. All three FP calls (one in patient 1 and two in patient 2) we observed belong to this class. Given ~50 Mb genomic regions were targeted for resequencing, one or two FPs corresponds to a FP rate of 0.02-0.04 per Mb. The actual FP rate could be a little higher because we did not resequence every HC mutation for validation, but this number is well below 0.16 per Mb (the FP rate for muTect [23]), supporting that our procedures indeed guards against FPs as compared to the state-of-the-art mutation callers. Regarding the false discovery rate (FDR), 1.1% (2/184) to 1.6% (1/62) of all HC mutations could be FP. We conclude that miscalling homozygous reference genotype in tumor DNA is the major source of FP calls.

FN calls of a shared mutation might decrease the power of clonal status test, causing a Type II error. A FN arises when there is truly a shared mutation but it is erroneously missed in either one or both tumors. We could imagine that low cellularity in both tumors could result in missing an unknown number of shared mutations in both tumors. However it is impractical to estimate the chance for such event since no gold standard exists for a complete set of shared mutations. But it should be less harmful to the CLS than missing a shared mutation in just one tumor because it simultaneously decreases the numerator and denominator. Hence we focused on the other possibility of misidentifying shared mutations as private mutations. We observed a moderately high number of FNs in patient 1 with one tumor of low cellularity and the other of moderate cellularity, where 8 of 51 validated shared mutations were erroneously called as private. This is in contrast to patient 2 with both tumors having moderate cellularity. For patient 2, we investigated the most likely candidates across the exome for FN calls, and selected all sites with an alternate allele with prevalence of at least 5% in both tumors. None were among the HC mutations, and all were validated as true negative calls. Therefore, we conclude that low cellularity in only one but not the other tumor seems the major source of FN calls.

We note that FN calls for shared mutations (which lessen the numerator) as well as false private mutation calls (which enlarge the denominator) can both decrease the observed CLS and thus the effect size. A stringent caller like the one we used in this study should make fewer false private mutation calls. Regarding the FN calls in a clonal pair, mutations may fail to be shared because of a FN call in one tumor or not the other and this was the major source of error in our data. This usually happens when one tumor but not the other is of low cellularity. At the first glance, using a more sensitive mutation caller like MuTect which has the potential to detect additional true somatic mutations in the low cellularity tumor seems a solution (though always at the price of increasing the type I error). However, it turns out that in the SBBC test setting, a more sensitive caller does not necessarily help increase the effect size, because calling shared mutations requires a balanced sensitivity in both tumors regardless of their cellularity. Furthermore, at the alternate allele fraction below 7.5% for which our callers started to generate FN calls, it is already very challenging to distinguish clonal from subclonal mutations at the standard read depth for exome sequencing. It is worth noting that the clonal status test works best for mutations which are present in a majority of tumor cells. Targeting sub-clonal mutations in the test is not recommended because it this might result in both elevated FPs and FNs. A more sensitive caller might call more false private mutations and/or sub clonal mutations, further reducing the CLS. This consideration justifies using a moderately instead of a highly sensitive mutation caller in this setting.

**References**

1. Li H, Durbin R. Fast and accurate short read alignment with Burrows-Wheeler transform. Bioinformatics. 2009;25(14):1754-60.

2. DePristo MA, Banks E, Poplin R, Garimella KV, Maguire JR, Hartl C, et al. A framework for variation discovery and genotyping using next-generation DNA sequencing data. Nat Genet. 2011;43(5):491-8.

3. Koboldt DC, Zhang Q, Larson DE, Shen D, McLellan MD, Lin L, et al. VarScan 2: somatic mutation and copy number alteration discovery in cancer by exome sequencing. Genome Res. 2012;22(3):568-76.

4. Olshen AB, Venkatraman ES, Lucito R, Wigler M. Circular binary segmentation for the analysis of array-based DNA copy number data. Biostatistics. 2004;5(4):557-72.

5. Nilsen G, Liestol K, Van Loo P, Moen Vollan HK, Eide MB, Rueda OM, et al. Copynumber: Efficient algorithms for single- and multi-track copy number segmentation. BMC Genomics. 2012;13:591.

6. Bao L, Pu M, Messer K. AbsCN-seq: a statistical method to estimate tumor purity, ploidy and absolute copy numbers from next-generation sequencing data. Bioinformatics. 2014. Epub 2014/01/07.

7. Cingolani P, Platts A, Wang le L, Coon M, Nguyen T, Wang L, et al. A program for annotating and predicting the effects of single nucleotide polymorphisms, SnpEff: SNPs in the genome of Drosophila melanogaster strain w1118; iso-2; iso-3. Fly. 2012;6(2):80-92.

8. Li H, Handsaker B, Wysoker A, Fennell T, Ruan J, Homer N, et al. The Sequence Alignment/Map format and SAMtools. Bioinformatics. 2009;25(16):2078-9.

9. Cibulskis K, Lawrence MS, Carter SL, Sivachenko A, Jaffe D, Sougnez C, et al. Sensitive detection of somatic point mutations in impure and heterogeneous cancer samples. Nat Biotechnol. 2013;31(3):213-9.
